# Novel multielectrode mapping catheter for targeting left ventricular summit derived premature ventricular contractions within the great cardiac vein: first-in-man

**DOI:** 10.1093/ehjcr/ytaf254

**Published:** 2025-05-22

**Authors:** Christian-H Heeger, Henning Rolfes, Martin W Bergmann, Maxim Didenko

**Affiliations:** Department of Rhythmology; Cardiology and Internal Medicine, Asklepios Klinik Hamburg Altona, Paul-Ehrlich-Straße 1 Hamburg 22763, Germany; German Center for Cardiovascular Research (DZHK), Partner Site Hamburg/Kiel/Lübeck, Lübeck, Germany; Department of Rhythmology, University Heart Center Lübeck, University Hospital Schleswig-Holstein, Ratzeburger Allee 160, 23538 Lübeck, Germany; Department of Rhythmology; Cardiology and Internal Medicine, Asklepios Klinik Hamburg Altona, Paul-Ehrlich-Straße 1 Hamburg 22763, Germany; Department of Rhythmology; Cardiology and Internal Medicine, Asklepios Klinik Hamburg Altona, Paul-Ehrlich-Straße 1 Hamburg 22763, Germany; Clinic for Electrophysiology, Herz- und Diabeteszentrum NRW, Ruhr-Universität Bochum, Med. Fakultät OWL (Universität Bielefeld) Bad Oeynhausen, Georgstr. 11, D-32545 Bad Oeynhausen, Germany

## Case description

**Figure 1:**
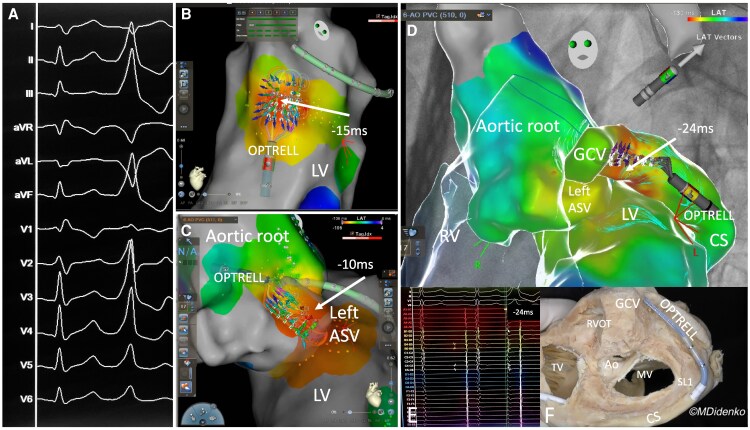
(*A*) Twelve lead electrocardiogram with frequent monomorphic premature ventricular contractions: Inferior axis, positive concordant chest leads, a slurre QRS-complex onset and a QS configuration in lead I: suggestive for an origin within the left ventricular-summit. (*B*) OPTRELL mapping catheter during 3D mapping (Carto 3; V8.2, J&J MedTech) of the left ventricular-summit. Please notice the conduction velocity vectors (colourful arrows) diverting from the center of the grid of the OPTRELL catheter precisely detecting the origin of the premature ventricular contractions (−15 ms preceding QRS-complex onset, earliest activation time). (*C*) OPTRELL mapping catheter during 3D mapping of the aortic root within the left coronary cusp. Please notice the conduction velocity vectors (colourful arrows) diverting from the left coronary cusp precisely detecting the origin of the premature ventricular contractions (−10 ms preceding QRS-complex onset, earliest activation time). (*D*) Merged 3D map of right ventricle, left ventricular, aortic root and coronary sinus/great cardiac vein: OPTRELL mapping catheter during 3D mapping within the great cardiac vein. Please notice the conduction velocity vectors (colourful arrows) diverting from the great cardiac vein precisely detecting the origin of the premature ventricular contractions. The target ablation area lies in the triangle between left coronary cusp, left ventricular, and great cardiac vein. The successful ablation site was in the distal great cardiac vein. (*E*) Intracardiac electrograms from the HDME catheter with the earliest activation time (−24 ms preceding QRS-complex onset). (*F*) Corresponding anatomical specimen with the OPTRELL mapping catheter within the distal great cardiac vein via a SL1 sheath. LCC, left coronary cusp; RV, right ventricle; GCV, great cardiac vein; PVC, premature ventricular contractions; LV, left ventricular.

A 20-year-old male patient presented with symptomatic monomorphic premature ventricular contractions (PVC) (34% PVC burden). The 12 lead electrocardiogram showed an inferior axis, positive concordant chest leads, a slurred QRS-complex onset and an QS configuration in Lead I, suggestive for an origin within the left ventricular (LV)-summit.^1,2^ The patient was scheduled for a catheter ablation procedure with a novel high density multielectrode (HDME) (*n* = 36 electrodes) grid-shape mapping catheter (OPTRELL, J&J MedTech). After transseptal puncture the mapping catheter was positioned at the endocardial LV-summit and a 3D map was generated (Carto 3; V8.2, J&J MedTech) with an earliest activation of 15 ms preceding QRS-complex onset. Additionally, the aortic root was mapped with an earliest activation of 10 ms at the left sinus of valsalva.^3^ Due to the slurred QRS-complex onset and insufficient activation an epicardial origin was suspected and the OPTRELL was positioned within the distal coronary sinus and was further advanced to the great cardiac vein (GCV) via a SL1 sheath. High density multielectrode mapping revealed the earliest activation at the distal GCV, preceding the QRS-complex onset by 24 ms. A contact force sensing ablation catheter (STSF, J&J MedTech) was advanced to the GCV and irrigated ablation (17 mL/min, 25W, AI 600) was performed. The PVC terminated immediately and the patient was discharged on the next day without any issues. With HDME mapping PVC beats can be captured automatically and activation and conduction can be visualized live by each individual electrode. This ability might be an advantaged to conventional mapping strategies. Although manoeuvring the HDME catheter to the GCV was feasible without any complications in this single case operators should be cautious. Here we are presenting the first case report of an epicardial LV-summit derived PVC targeted via the GCV. The PVC was rapidly and precisely located and targeted utilizing a novel HDME grid shaped mapping catheter suggestive for a feasible approach (*Figure 1*).

**Consent:** The patient consent was obtained.

## Data Availability

The data underlying this article will be shared on reasonable request to the corresponding author.
